# Effect of Starch Type and Pre-Treatment on the Properties of Gelatin–Starch Foams Produced by Mechanical Foaming

**DOI:** 10.3390/polym15071775

**Published:** 2023-04-02

**Authors:** Virginia Martin Torrejon, Hang Song, Bingjie Wu, Guidong Luo, Jim Song

**Affiliations:** 1 Media and Communication School, Shenzhen Polytechnic, Shenzhen 518055, China; 2 School of Innovation and Entrepreneurship, Southern University of Science and Technology, 1088 Xueyuan Avenue, Shenzhen 518055, China; 3 Centre for Food Policy, Myddelton Street Building, City University of London, London EC1R 1UW, UK

**Keywords:** starch-based bioplastics, gelatin-based cellular solids, bio-based packaging foams, mechanical foaming of biopolymers, renewable packaging, cushioning

## Abstract

Incorporating biopolymers in packaging foams can contribute to a more circular packaging system, utilizing renewable and compostable materials. Gelatin, with its favorable physicochemical properties, allows for producing gelatin foams via mechanical foaming, a well-established and low-investment process. To improve foam properties, starch can be added to the gelatin formulation. However, the variability in the properties of starch powders can impact the polymer blend and, consequently, the properties of the dry foam. This study aimed to investigate the impact of different starch powders from different botanical origins (tapioca and corn) and treatments (native or pregelatinized) on the properties of gelatin–starch foams produced by mechanical foaming. The study successfully produced foams with densities of approximately 45–50 kg/m^3^ and compression properties comparable to EPS (expanded polystyrene) foams. The starch type and pre-treatment significantly influenced the properties of the foam. Pregelatinized starches exhibited slightly higher densities due to lower foamability caused by higher viscosity. Using starch exhibiting total loss of birefringence led to denser foams with greater compression properties than those with starch with a certain degree of crystallinity remaining. Therefore, selecting the appropriate starch type is crucial when developing starch-based materials to ensure optimal material and processing properties align with application requirements.

## 1. Introduction

Foams are cellular solids that can be found in many forms, both naturally and through human-made materials. Natural foams include those found in animal bone structures, marine organisms such as sponges and plant materials such as cork. Meanwhile, foams made from ceramics, metals and plastics are commonly used in various industrial applications. Plastic packaging foams are widely used for different applications, including food containment and thermal/mechanical protection for the delivery of food products. These plastic foams exhibit excellent properties (low density, low thermal conductivity, high impact strength and low cost) and are mainly made of petroleum-derived feedstocks, such as polystyrene (PS), polyethylene (PE) and polyurethane (PU). However, while these are recyclable materials, a considerable amount of plastic packaging waste leaks from the waste management systems and pollutes our ecosystems [1]. 

The principles of the circular economy promote resource efficiency and waste reduction by keeping materials in use for as long as possible. Utilizing renewable and compostable biopolymers in packaging foams aligns with these principles, providing a sustainable and resource-efficient system that reduces waste and promotes long-term environmental sustainability. Additionally, biopolymers tackle the biocompatibility issues raised by conventional plastics [2] and are readily available and relatively inexpensive [3]. Thus, biopolymers offer a promising alternative to conventional plastics in terms of functionality and economics, and by carefully selecting the appropriate biopolymer and adjusting processing conditions, biopolymer foams can achieve properties and performance competitiveness with conventional plastics [4].

In recent years, considerable research has been conducted on developing biofoams for packaging applications. Starch-based foams have been extensively researched as an alternative to conventional plastic foams, with different processing methods, such as extrusion and baking, yielding promising results [5,6,7,8]. Mycelium-based foams have also emerged as a promising area of research for sustainable packaging solutions, offering biodegradability, compostability and comparable properties to traditional petroleum-based foams [9,10,11]. In addition, extensive research has been conducted to enhance the properties and sustainability of foams by developing biopolymer blends and composites. Natural fibers and nanocellulose have emerged as promising materials to incorporate into foam formulations. Biocomposite foam materials containing natural fibers, such as kenaf, hemp and flax, have improved mechanical strength, modulus and thermal stability [12,13,14,15,16,17,18]. Nanocellulose, on the other hand, is a valuable nanofiller for biopolymer foam composites, offering potential improvements in foam properties and cost reduction [19,20].

However, implementing biofoams as bioplastics has encountered several challenges, including their high cost compared to their fossil fuel counterparts, low manufacturing efficiency and uncertainty on how to dispose of them after use [21]. Thus, developing novel competent biofoams must integrate the investigation of cost-effective formulations and processing methods capable of producing materials comparable to conventional plastics with a clear EOL (end-of-life) option. While some commercially available biobased foams have been established, such as starch-based loosefill used for product cushioning during transportation, there is still no strong alternative to compete with PS and PE in plank/sheet formats. Therefore, there is a need to develop cost-effective biofoams for packaging applications in bulk as an alternative to conventional plastics.

Gelatin is a by-product of the food industry used and investigated for numerous applications, such as biomedical products, packaging or tissue engineering. Gelatin is an ideal candidate for biomaterials development due to its outstanding properties: relatively easy processability, availability, low cost, biocompatibility and biodegradability [22]. In addition, gelatin physicochemical properties (e.g., low surface tension, low viscosity, sol-gel behavior, tailored gel strength by using different gelatin types and content) facilitate the production of gelatin foams through different processing methods [23]. Among these production methods, the mechanical foaming of gelatin solutions allows the production of stable and high-expansion foams by taking advantage of the gelatin solution’s rapid gelation and stabilization. In addition, mechanical foaming is a well-established process that does not require extensive equipment investment and development. Formulation-wise, gelatin-derived foams can benefit from incorporating additives that can optimize their properties or reduce costs, such as lignocellulosic fillers or starch. Starch is an inexpensive and widely available carbohydrate used in packaging materials formulations as the main biopolymer or an additive/filler [24,25,26] that has been used to enhance gelatin films’ properties [27]. However, starch powders from different botanical origins, growing characteristics and treatments are expected to exhibit different physical and chemical properties, such as granule morphology, gelatinization temperature, relative crystallinity and amylose content [28,29]. These properties’ disparity between starches can impact the biofilm properties (e.g., density and mechanical properties) made by different processing methods [30]. Thus, when using starch powders for biofoams formulation development, understanding the impact of the starch type on the gelatin foam properties is essential to achieve foam products competitive with conventional plastics in functional and economic terms. Thus, this work aimed to produce gelatin-based foams by mechanical foaming using a lab-scale production line and to investigate the incorporation of native and pregelatinized starch powders from two botanical origins (tapioca and corn) to identify the characteristics of the starch powders producing the most desirable foams for packaging applications.

## 2. Experimental Details

### 2.1. Materials

Gelatin powder (Type B, 240 Bloom, average molecular weight (Mw) 122,400 g/mol, isoelectric point 4.8, Dongbao Bio-Tech Co. Ltd., Baotou, China) is from a mixture of cow and pig bones. The moisture content of the as-received material was 11%, measured with a moisture analyzer (HE77, Mettler Toledo, Columbus, OH, USA).

Four commercial starch powders were used: native tapioca (NT), pregelatinized tapioca (PT), native corn (NC) and pregelatinized corn (PC) starches. NT, PT and NC starches (Guangzhou Hongyi Chemical Co. Ltd., Guangzhou, China) and PC starch (Qufu Tianli Pharmaceutical Excipients Co. Ltd., Qufu, China) were supplied by different suppliers. The native-pregelatinized starch powder pairs studied here did not come from identical harvesting batches; thus, while they come from the same species, inevitable variability in physicochemical properties is expected due to harvesting, climatic and genotypic differences [28]. Table 1 shows the chemical composition of the four starches and the gelatin.

Sodium dodecyl sulfate (SDS) (assay 98.5%, BioFroxx, Einhausen, Germany) and deionized water was used as a solvent. Tap water was used as the primary source of water for all experiments. Tap water was chosen due to its availability and low cost and its comparability to the type of water typically used in the community where the research was conducted.

### 2.2. Sample Reparation 

Table 2 shows the composition of the six formulations studied in this work. The SDS content was kept constant at 0.75 wt.% of the total gelatin–starch suspension for all the samples. These formulations were selected from preliminary investigations aiming to achieve low-density dry foams with desirable mechanical properties and virtually no shrinkage. 

The preparation of the foams consisted of the following stages: (a) Thermoplastic starch (TPS) preparation, if required, (b) additives mixing (gelatin and SDS), (c) mechanical foaming with gas injection, (d) casting and (e) drying. In terms of the physical state of the materials, the process consisted of four stages: (a) liquid stage (during the additives mixing stage), (b) liquid foam stage (after the mechanical foaming stage), (c) gel foam (following the casting stage) and (d) dry foam (once the drying process is finalized).

#### 2.2.1. Thermoplastic Starch Preparation

It is necessary to disrupt the crystalline starch structure by heating and shearing treatment to obtain an amorphous paste suitable for materials processing, the so-called thermoplastic starch (TPS). Thus, the starch powders were heated and mixed in water with a weight ratio of 1/10 (starch/water) at 80 °C for 20 min in a stirring reactor (custom-made, Hangzhou Wangge Mechanical Equipment Ltd., Hangzhou, China) at a 50 Hz stirring frequency. The starch suspension was then sheared at 80 °C for 10 min with an electric hand blender with a blade accessory (MQ787, Braun Frankfurt, Frankfurt am Main, Germany) until a homogeneous starch paste was obtained. 

#### 2.2.2. Foams Preparation

The gelatin, SDS, water and the TPS (if required) were added to the stirring reactor and mixed for 30 min at 60 °C. The mixtures were then foamed with a foam generator (WG Series, Hangzhou Wangge Mechanical Equipment Ltd., Hangzhou, China) at a liquid flow rate of 50 kg/h, 1000 rpm stirring speed and 45 °C. After foaming, the liquid foams were immediately cast in 15 × 15 × 5 cm rubber molds and let dry in an environmental chamber at 23 °C and 20% RH. Once dried, the foams were stored for at least 21 days under controlled conditions (23 °C and 50% RH) before characterization, when the degree of retrogradation of starch is expected to plateau [31].

### 2.3. Characterization of the Starch Powders

#### 2.3.1. Crystallinity

The X-ray diffraction (XRD) spectra of the starch powders were recorded using a diffractometer (6100, Shimadzu, Kyoto, Japan) with Kα copper radiation, 40 kV voltage and 20 mA current. Assays were performed for 2θ between 3 and 75° with steps of 0.05 °/s. The XRD patterns were truncated to the 4–35° region of the diffraction angle (2θ) and smoothed by applying the Savitzky–Golay filter with a polynomial of degree equal to 3 and 20 points. The linear baseline correction was then applied to the data points of the selected region, offsetting to a zero-intensity value and intensity normalized between 0 and 1 of arbitrary units (AU). The degree of crystallinity was estimated using the method proposed by Nara and Komiya [32], calculated as the ratio between the area corresponding to the crystalline phase and the total area under the XRD.

#### 2.3.2. Morphology

The morphology of the starch powders was studied through a Scanning Electron Microscope (SEM) (Volume Scope 2, Thermo Fisher Scientific, Waltham, MA, USA) operating at an accelerating voltage of 5 kV. The samples were coated with a thin layer of gold using a sputter coater (Q150R S plus, Quorum Technologies, Laughton, UK) to minimize electrical charge during observation.

### 2.4. Characterization of the Suspensions

#### 2.4.1. Differential Scanning Calorimetry (D.S.C.)

DSC measurements were conducted using a differential scanning calorimeter (Q20, TA Instruments, New Castle, DE, USA). Approximately 3 mg of starch/water suspensions with 10 wt.% starch content were placed in a hermetically sealed aluminum pan and equilibrated for 1 h at 23 °C. The pan was then ramped from 23 °C to 100 °C at a constant heating rate of 10 °C/min under a nitrogen atmosphere (50 mL/min) in a single cycle to determine the glass transition temperature of the suspensions.

#### 2.4.2. Microscopic Assessment of the T.P.S. Preparation Process

The starch powders in a 10 wt.% water solution before thermal and mechanical treatment and the starch pastes after TPS processing were observed by an optical microscope (Zeiss Axio, Carl Zeiss Microscopy GmbH, Jena, Germany) using a regular and polarized light filter equipped with a digital camera. 

#### 2.4.3. Viscosity 

The viscosity of the formulated liquids was measured at 45 °C using a rheometer (Haake Mars III, Thermo Fisher Scientific, Waltham, MA, USA) equipped with a Peltier temperature control system. The solutions were loaded into a 45 °C pre-heated double gap cup (3 mL capacity) and stabilized for 60 s. The measurements were performed using an isothermal shear rate ramp test (45 °C) from 0.01 s^−1^ to 1000 s^−1^ with 60 measuring points in 600 s. 

#### 2.4.4. Surface Tension 

The surface tension of the formulated liquids was measured at 45 °C by the Wilhelmy plate method using a tensiometer (K100, Krüss, Hamburg, Germany). 

### 2.5. Characterization of the Gels

The strength of the gelatin and gelatin–starch gels was measured using a texture analyzer (TA.GEL, Bosin, Shanghai, China). The formulated gelatin and gelatin–starch solutions were prepared according to the Bloom test method, typically used to measure the strength of gelatin gels [33]. The gels were transferred to a water bath at 10 °C and kept for 17 h before measurement. The strength measurement was obtained according to the force in grams needed by a cylindrical plunger with a diameter of 0.5 inches to depress the surface of the gel by 4 mm.

### 2.6. Characterization of the Foams

#### 2.6.1. Expansion Ratio

The expansion ratio of the liquid foams was calculated using Equation (1) [34], where ER is the expansion ratio obtained after mechanical foaming and *ρ_l_* and *ρ_lf_* are the density of the liquid before foaming and the density of the liquid foam immediately after foaming, respectively.

(1) $$ER=\frac{\rho_{lf}}{\rho_{l}}$$

#### 2.6.2. Bulk Density, Relative Density and Porosity

The dry foam bulk density (ρ*) was determined according to the ISO 845:2006 standard by calculating the ratio between the mass of a dry foam sample and its volume. At least five specimens from each formulation were measured. The relative density (*ρ_r_*) was calculated by dividing the bulk density of the foam by that of the dry solid from which the cell walls were made (*ρ_S_*), which was obtained by casting the gelatin and gelatin starch gels and calculating the ratio between their weight and volume, measured by the glass bead displacement method. Finally, the porosity of the solid foam (P) was determined from Equation (2) [35]:

(2) $$P=1-\rho_{r}$$

#### 2.6.3. Foam Structure

The cellular structure of the foams was determined using an optical microscope (Stemi 508, Carl Zeiss Microscopy GmbH, Germany). The camera was set up at 2592 × 1944 pixels. The surface of each specimen was colored using black ink to facilitate the visualization of the cellular structure. The mean pore area (an average of 600 measurements), mean cell area (an average of 300 measurements) and cell struts thickness of the foams were calculated using the ImageJ software. 

#### 2.6.4. Compression Properties

The compression tests of the foams were conducted according to the ASTM D-1621 standard. A universal testing machine (Instron Universal Testing Machine, Instron, Norwood, MA, USA) with a 10 kN-load cell and a crosshead speed of 2.5 mm/min was used to characterize square foam specimens with a 100 cm^2^ area and 25.5 mm height. The compressive strength and Young’s modulus were derived from the stress–strain curves averaging the measurement results of five specimens. In addition, the foams’ elastic and plastic energy absorption was calculated by integrating the area under the stress–strain curve at the yield strain (or 10% strain if the sample did not exhibit a yield point) and 50% strain, respectively. The compression properties of commercial EPS cushion packaging blocks of 20 and 30 kg/m^3^ were also measured for comparison.

### 2.7. Statistical Analysis

Analysis of variance (ANOVA) with (*p* = 0.05) was performed using Minitab 17.1.

## 3. Results and Discussion

### 3.1. Thermoplastic Starch (TPS)

This research aimed to design a continuous mixing and foaming process, firstly performing the TPS preparation and, secondly, adding and mixing all the additives included in the formulation of the foams to the TPS paste.

**Figure 1:**
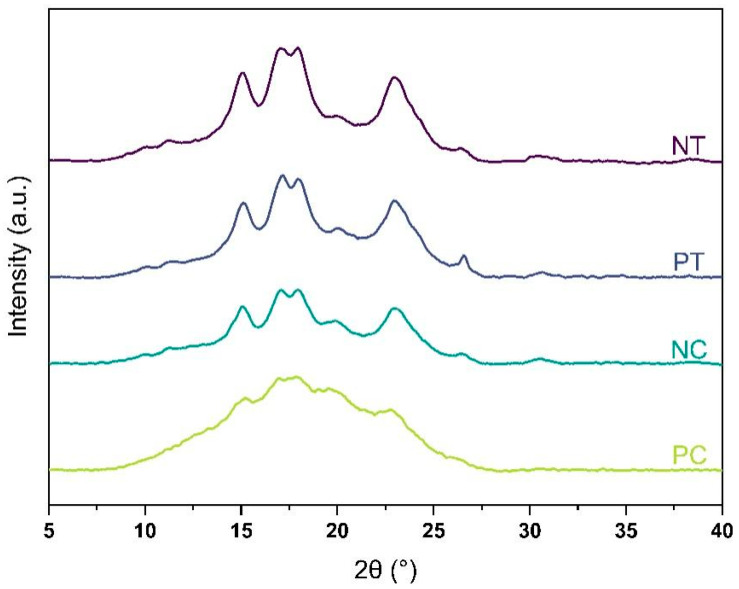
X-ray diffraction patterns of the four starches: NT, native tapioca; PT, pregelatinized tapioca; NC, native corn; PC, pregelatinized corn.

Starch is a type of polysaccharide that consists of both crystalline and amorphous phases. Its level of crystallinity, which typically ranges from 14 to 45%, is determined by the relative amounts of amylose and amylopectin present in the starch granules [20]. The presence of both crystalline and amorphous phases in the four starch powders studied was confirmed by XRD analysis. However, as depicted in Figure 1, the intensity of XRD peaks was weaker for pregelatinized starches, indicating lower crystallinity degrees compared to native starches.

The degree of crystallinity was 36% and 34% for native tapioca and corn starches, respectively, while it was 26% and 9% for pregelatinized tapioca and corn starches, respectively (see Table 3). These findings imply that while pregelatinized corn starch can be considered predominantly amorphous, the pregelatinized tapioca treatment from the supplier was not as effective as expected due to its high degree of crystallinity.

**Figure 2:**
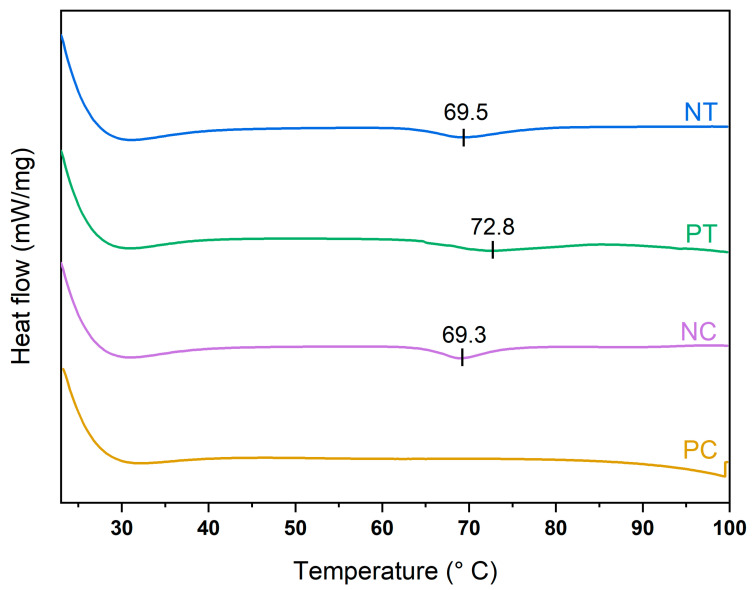
DSC graphs of the four starches: NT, native tapioca; PT, pregelatinized tapioca; NC, native corn; PC, pregelatinized corn.

The disruption of the crystalline starch structure by thermal treatment was investigated by studying the DSC thermal transitions at 10 wt.% starch content dispersions. The starch–water dispersions showed the typical DSC endothermic transition between 60 °C to 80 °C with well-defined onset (T_0_), peak (T_p_) and concluding (T_c_) transition points in the native starches (see Figure 2), as similarly found in the literature [36]. This DSC endotherm involves the swelling of granules’ amorphous regions and the melting crystallites. T_p_ typically represents the water movement into the amorphous regions of the starch granules. In contrast, the starch structure disruption mainly occurs at temperatures closer to T_c_ [37]. Thus, 80 °C was selected as the processing temperature for disrupting the starch granules, a relatively low value to minimize energy consumption during processing.

**Figure 3:**
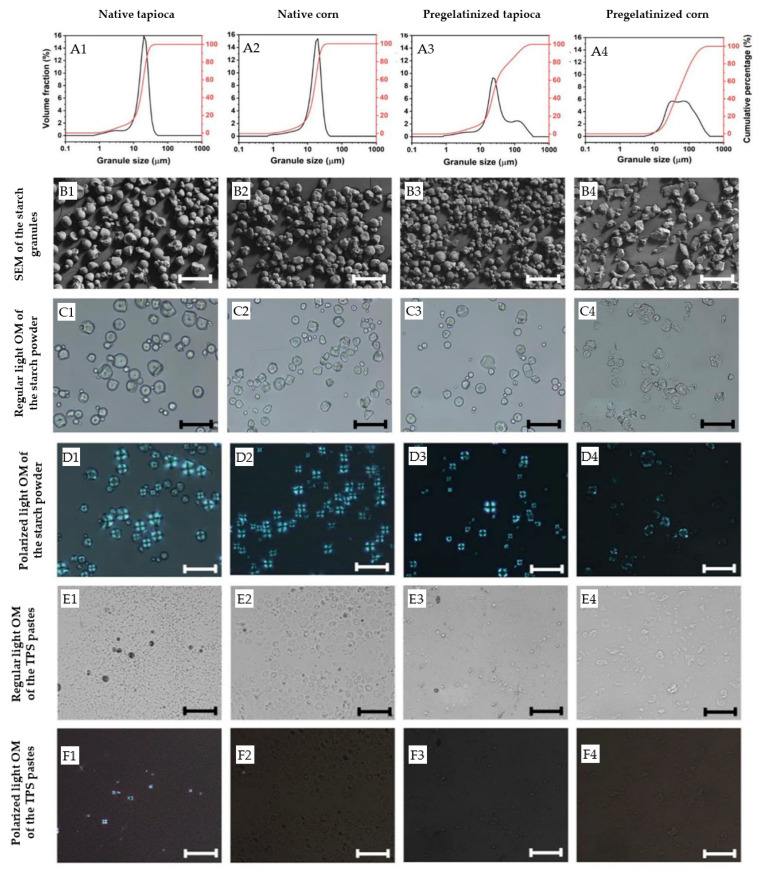
(**A1**–**A4**) Granule size distribution and morphology of the starch granules under (**B1**–**B4**) SEM, and (**C1**–**C4**) regular and (**D1**–**D4**) polarized light optical microscope (OM) images of the starch granules and (**E1**–**E4**) regular and (**F1**–**F4**) polarized light optical microscope (OM) images of the starch granules the pastes after TPS processing. Scale bar = 50 μm.

Microscopy images of the starch pastes after TPS treatment are shown in Figure 3(E1–E4), which reveal that shearing and heating at 80 °C disrupted the starch granules. The granule size decreased significantly in all four starches after treatment, as seen when compared with Figure 3(C1–C4). However, some granule ghosts remained embedded in the TPS pastes. Polarized microscopy (PM) analysis of the TPS pastes (Figure 3(F1–F4)) showed a total loss of birefringence of the starch granules (see Figure 3(D1–D4)), indicating starch gelatinization in the native corn and the pregelatinized tapioca starches, which was accompanied by an increase in viscosity due to granule swelling and amylose leaching. The native tapioca still exhibited some granules with structural order, implying partial gelatinization, probably due to insufficient thermal treatment. It is worth noting that Tc, which is assumed to represent the gelatinization temperature of starch granules, does not always refer to the total gelatinization of starch [37] as gelatinization is a broad range temperature transition, rather than a narrow temperature event [38]. In addition, the native tapioca was slightly less viscous than the other starch powders, which may have had a detrimental effect on the shear stress on the melt [39]. Therefore, higher processing temperatures and shearing stress should be applied for future work to process the native tapioca starch investigated here.

Figure 3 also highlights the significant impact of starch pre-treatment from the supplier on granule size distribution. Due to granule agglomeration, pregelatinized starches exhibited slightly larger granule sizes (see Table 3). The distribution curves of the four starch types displayed a slight shoulder representing small granules or fragments (see Figure 3(A1–A4)). However, while the granule size of the native starches exhibited a unimodal size distribution ranging from approximately 1 to 50 μm, the pregelatinized starches displayed a bimodal size distribution with their small particles ranging from around 1 to 50 μm and their larger particles ranging from 300 μm to 400 μm due to the formation of agglomerates. Other studies have also reported a widening in granule size distribution due to thermal treatment [36], which can lead to processing difficulties such as longer rehydration rates and agglomeration during redispersion and mixing [40]. However, no such processing difficulties were observed during the mixing process of any of the starches investigated in this study.

### 3.2. Gelatin-TPS-SDS Suspensions and Gels

The suspensions were prepared by mixing TPS with gelatin, maintaining a 25 wt.% solid content (20 wt.% gelatin content and 5 wt.% starch content), and their viscosity, surface tension and foamability (i.e., expansion ratio) were investigated and compared to those from 20 wt.% and 25 wt.% gelatin content solutions. The foaming parameters (i.e., stirring speed and liquid and gas flow rates) were optimized and standardized to create monodisperse and stable high-expansion foams. A foaming temperature close to the dispersions’ gelling point [41] was selected to achieve fast liquid foam stabilization by arresting the liquid foam shrinkage by rapidly gelling the gelatin-TPS matrix [42].

As seen in Table 4, all the suspensions exhibited relatively low surface tension due to the high surface activity of gelatin solutions [41]. However, the pure gelatin solutions displayed lower viscosities and surface tension and, consequently, higher expansion ratios than those containing starch. Regarding the starch–gelatin dispersion, the starch type slightly influenced their expansion ratio (*F*_3–16_ = 6.99, *p* = 0.0032), mainly determined by the liquid’s viscosity (*F*_3–16_ = 35.76, *p* < 0.001) and surface tension (*F*_3–16_ = 7.97, *p* = 0.0018). The suspensions prepared from native tapioca achieved the highest expansion ratio after mechanical foaming (10.31), mainly due to their relatively low viscosity (1768 mPa·s), the lowest among the ones made with starch. On the other hand, the lowest expansion ratio corresponded to the pregelatinized corn suspensions (8.88), which also displayed the highest viscosity among the studied dispersions. 

These results proved that slight viscosity and surface tension changes between formulated liquids lead to slight differences in expansion ratios. Higher expansions are obtained from liquids that facilitate the gas incorporation into the matrix, allowing the gas bubbles to expand and stretch. Thus, liquids with higher viscosities require higher energy to create enough shearing to introduce air into the liquid, limiting the capacity to achieve higher air volume fractions in the liquid foam. Higher energy in the process involves the modification of the foaming parameters, including faster stirring speeds and higher foaming temperatures, but the study of their impact on the liquid foams’ properties is beyond the scope of this paper.

As expected, the gels prepared with higher gelatin concentrations produced stronger gels [23]. The type of starch used also considerably affected the gel strength of the gelatin–starch gels (*F*_3–16_ = 49.27, *p* < 0.001), with those prepared with pregelatinized tapioca being the strongest and those prepared with pregelatinized corn the weakest. In addition, the effect of incorporating starch into formulations with 20 wt.% gelatin decreased its gel strength for native tapioca and pregelatinized corn and increased it for pregelatinized tapioca and native corn.

### 3.3. Gelatin–Starch Foams 

#### 3.3.1. Density 

Bulk density and ER exhibited a negative linear correlation (r = −0.9825), and as expected, lower-density foams were achieved at higher ER as drying shrinkage was insignificant in all the formulations (<5 vol.%). The lowest density achieved, 30.21 kg/m^3^, was comparable to those achieved in conventional plastic foams used for packaging applications and corresponded to 20 wt.% gelatin foams, with an ER of 13.66 (see Table 5). 

The density of the gelatin foams considerably increased when their solid content increased from 20 wt.% to 25 wt.% due to the lower ER in the 25 wt.% gelatin solutions due to a considerable viscosity increase.

The starch type significantly affected the bulk density of the foams (*F*_3–16_ = 9.87, *p* < 0.001). Those foams made with tapioca starches had slightly lower bulk density than those made with corn starch: 44.03 kg/m^3^ (ER = 10.31) for native tapioca, 45.61 kg/m^3^ (ER = 9.60) for pregelatinized tapioca and 48.20 kg/m^3^ (ER = 9.42) and 49.45 kg/m^3^ (ER = 9.64) for native and pregelatinized corn, respectively. As expected, the pregelatinized starches produced foams with slightly higher densities than their native counterparts because their formulations were more viscous and led to lower ERs. 

While the gelatin and gelatin–starch foams exhibited a relatively higher density than packaging EPS foams (usually 20–30 kg/m^3^), they were reasonably light for packaging applications. In addition, while no attempt was made to optimize the foaming parameters further, there is scope for density reduction by modifying the mechanical foaming and the formulation parameters.

#### 3.3.2. Foam Structure

After mechanical foaming, the rapid gelation of the material composing the cell walls led to highly stable hydrogel foams for all the formulations investigated. Then, after drying, the hydrogel foams led to solid foams with minimum drying shrinkage (<5 vol.%).

**Figure 4:**
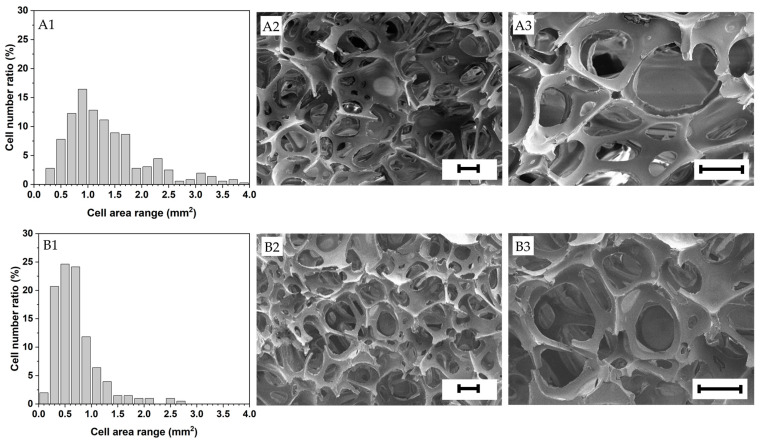
Cell area distribution and structure of the foams investigated (**A1**–**A3**) 20 wt.% gelatin, (**B1**–**B3**) 25 wt.% gelatin. Scale bar = 200 μm.

**Figure 5:**
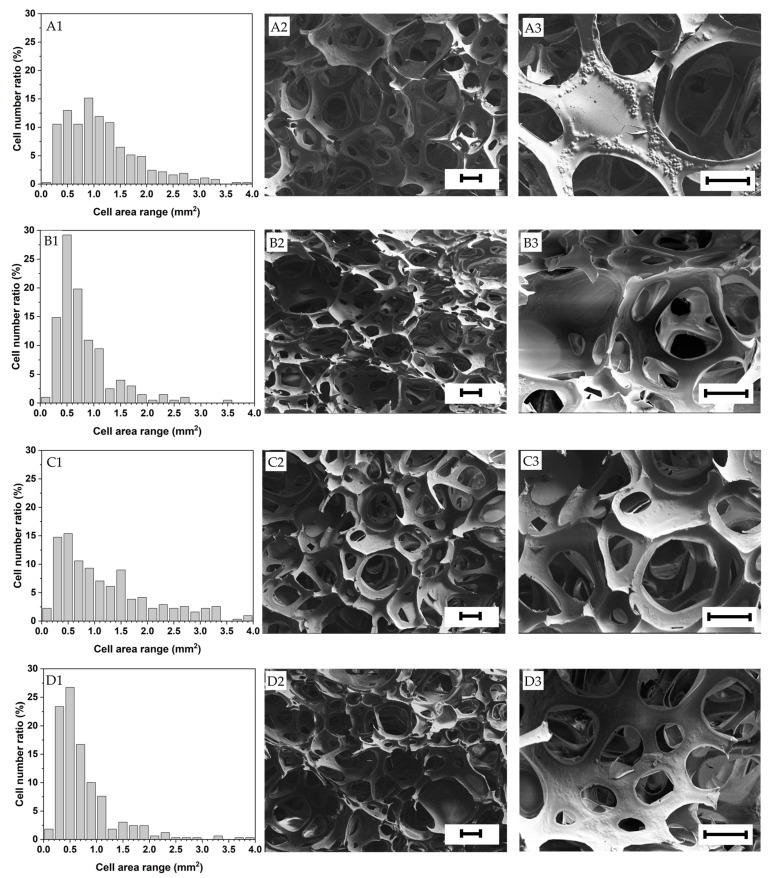
Cell area distribution and structure of the foams investigated (**A1**–**A3**) Native tapioca, (**B1**–**B3**) Pregelatinized tapioca, (**C1**–**C3**) Native corn, (**D1**–**D3**) Pregelatinized corn. Scale bar = 200 μm.

The foam structure is highly dependent on the expansion ratio of the suspensions and the factors arresting and delaying foam aging (i.e., drainage, coalescence and coarsening). All the foams investigated in this research exhibited a heterogeneous open cell structure with an average cell size ranging from approximately 0.5 to 1.5 mm and relatively wide cell size distributions (see Figure 4 and Figure 5). Gelatin foams prepared by mechanical foaming usually exhibit open cell structures due to the uncontrollable nature of air being introduced into them [43]. Open cell structures may have an advantage for materials requiring a drying process after foaming, like in this investigation, as the mass transport of moisture from the wet foam is facilitated, as opposed to close cell foams. 

Lower mean pore areas and narrower cell area distributions generally led to higher bulk densities. The 25 wt.% solid content foams made solely with gelatin presented the smallest mean cell area (0.775 mm^2^). This is attributed to their faster gelation than the rest of the foams due to their higher gelatin content (the other formulations had 20 wt.% gelatin content) [41], which speeded up foam stabilization and minimized foam aging. In contrast, the foams prepared with only gelatin at 20 wt.% exhibited the highest mean pore area (0.098 mm^2^) and a relatively high mean cell area due to exhibiting the highest expansion ratio. 

In addition, as shown in Table 6, the native starches displayed slightly larger mean pore areas (*F*_3–2396_ = 36.41, *p* < 0.001) and cell areas (*F*_3–1196_ = 15.83, *p* < 0.001) due to their lower viscosities that facilitated the gas incorporation into the solution and, thus, the elongation and growth of the foam cells. For the same reason, the foams made with the native starches also exhibited broader cell size distributions compared to the pregelatinized counterparts (Figure 5), being usually desirable to obtain foams with homogeneous mechanical properties and densities. For further details, optical microscopic pictures of the foam samples are shown in Supplementary Figure S1. 

The relics of the starch granules were evident on the dry foam cell walls, especially in the foams prepared with native starches (see Figure 5). The foams prepared with the pregelatinized starches displayed smoother cell walls, evidencing a better starch granule disruption.

#### 3.3.3. Compression Properties

The study of the compression properties of the foams aimed to assess their cushioning performance. The mechanical properties of foams depend on their density, structure and the material of the cell walls [35] and understanding their relationship can facilitate the optimization of the foam properties through processing and formulation adjustment. 

Compressive modulus, compressive strength and the deformation energy of the commercial EPS foams were comparable to the gelatin and gelatin–starch foams produced in this study. The 20 wt.% gelatin foams exhibited comparable mechanical properties to EPS 20 kg/m^3^, and the mechanical properties of 25 wt.% solid content foams were closer to the ones of EPS 30 kg/m^3^. However, in both cases, the foams produced in this study were slightly denser than the EPS foams. 

**Figure 6:**
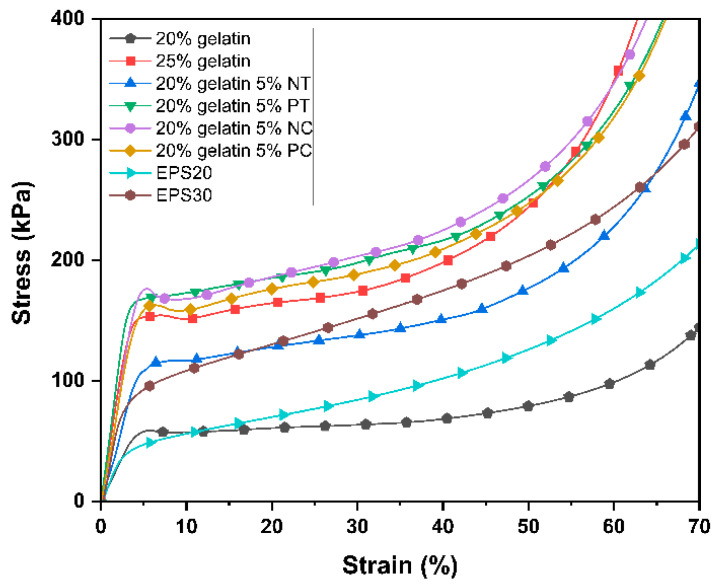
Comparison of typical compressive stress–strain curves (23 °C, 50% RH) for the gelatin and gelatin–starch foams produced by mechanical foaming.

Figure 6 shows the typical compressive stress–strain curves for the gelatin and gelatin–starch foams and the two commercial EPS packaging foams. The gelatin and gelatin–starch foams exhibited the characteristic stress–strain curves of elastomeric open cell foams, which consist of three distinct regions: (1) the linear elastic region, (2) the stress plateau region and (3) the densification region. The stress increased with strain in the linear elastic region, reaching a peak at approximately 5–7% strain, the yield point. For open-cell foams, the linear elastic mechanism primarily depends on cell walls bending up to the yield point at which they start to buckle and deform plastically [35]. The elastic deformation energy measures the elastic strain energy stored in the material. Compared to the EPS foams, the gelatin and gelatin–starch foams showed considerably lower deformation energy (see Table 5). In addition, the elastic region of the foams made with native corn starch was slightly narrower than the rest due to their greater rigidity.

The foams with 25 wt.% solid content exhibited a yield point followed by a slight stress decrease due to cell walls collapsing at the peak stress at the yield point. The 20 wt.% solid content gelatin foams and the EPS foams did not exhibit peak stress at the yield point due to a more progressive cell collapse due to having more flexible and thinner cell walls. As the load increased, the yield point was followed by the stress plateau, where cell buckling and collapse progressed at a relatively constant rate. Then, the foam densification stage started at approximately 40–50% strain, where the stress exponentially increased with the strain as the pores and the cells collapsed throughout the foam structure [35]. The foams should enter the densification region at high strain values to get less plastic deformation at normal loads in cushioning applications, as one of the main functions of polymeric packaging foam is to protect the packaged products by absorbing the impact energy during shipping and handling. The deformation energy at 50% strain is considered the energy absorption capability during the compression of the foams. Comparable deformation energy at 50% strain to the EPS foams (Table 7) was achieved for the foams made with native tapioca starch and solely with gelatin at 20 wt.% solid content exhibiting a lower rigidity.

Table 7 summarizes the mechanical properties obtained from the compression tests. The compressive modulus (E*) describes the elastic behavior of the foams in the elastic region, while the compressive strain and strength (σ*_el_) characterize the foam behavior as the cell structure collapses. The starch type significantly impacted the mechanical properties of the foams (F_3–16_ = 21.06, *p* < 0.001), the quality of the thermoplastic starch preparation process being the most significant factor. The foams prepared with native tapioca, whose TPS exhibited a certain degree of crystallinity manifested by remaining starch granules displaying the characteristic Maltese cross (Figure 2), showed the lowest compression modulus and strength. It is arguably known that the TPS treatment should be studied carefully and independently for each type of starch, as residual crystallinity can lead to inferior mechanical properties due to a less-coherent matrix of amylose and amylopectin [39,44]. Further work can consider processing native tapioca starch at a higher temperature. However, it was beyond the scope of this paper as it aimed to compare the performance of different starch powders using the same processing methods at minimum processing temperatures to maximize energy efficiency.

**Figure 7:**
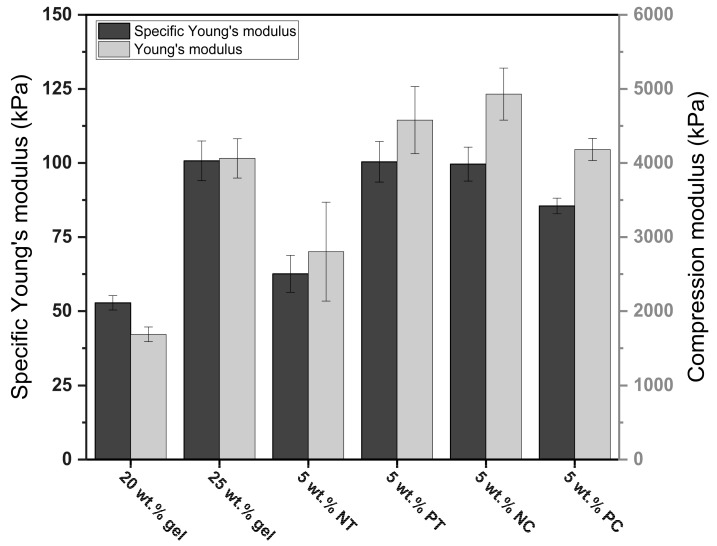
Comparison of the specific modulus and compression modulus of the gelatin and gelatin–starch foams.

In addition, the strength of the material from which the foams were made and the bulk density impacted the foams’ mechanical properties. Denser foams generally resist greater loads than light foams due to higher solid fractions and thicker cell walls [35], but this trend was not always evident in this investigation. However, as seen in Figure 7, the specific modulus of the 25 wt.% gelatin foams and those made with pregelatinized tapioca and native corn exhibited similar values, confirming the impact of bulk density on mechanical properties. However, the specific modulus of the foams containing pregelatinized corn was slightly lower than the other foams made with starch. This was attributed to the lower strength of their cell walls, but further research on the impact of gel strength and mechanical properties in gelatin-based foams is required.

**Figure 8:**
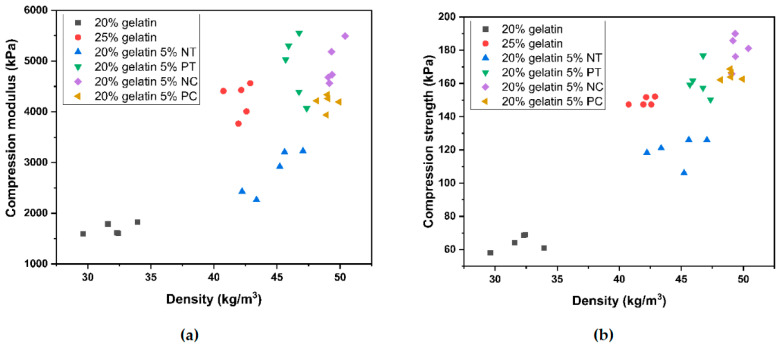
Scatter plot of (**a**) compressive strength and density and (**b**) compressive modulus (23 °C, 50% RH) for the gelatin and gelatin–starch foams produced by mechanical foaming.

Figure 8a,b show the compressive modulus and strength as a function of density for the gelatin and gelatin–starch foams. A strong correlation between foam density and compressive strength and modulus, regardless of the formulation, has been reported in other works [45]. However, while Figure 8 shows higher density foams generally exhibited higher modulus and strength, there is not a strong relationship between the two variables, indicating an evident influence of the matrix composition on the compression properties. 

## 4. Conclusions

Gelatin–starch foams with comparable compression properties to EPS foams used for packaging applications, yet with slightly higher densities, were successfully produced by incorporating different types of starch through mechanical foaming. The starch type and pre-treatment significantly impacted the foamability, surface tension and rheological properties of the starch–gelatin dispersions, which in turn affected their density and compression properties. Foams made with corn and pregelatinized starches showed slightly higher densities than those made with native and corn starch. Furthermore, the treatment process to produce thermoplastic starch (TPS) also significantly impacted foam properties. Foams made with TPS (pregelatinized tapioca, native corn, pregelatinized corn) exhibited total loss of birefringence and resulted in denser foams with higher compression properties. However, foams made with native tapioca displayed a certain degree of crystallinity and showed the lowest compression modulus and strength. Thus, a careful selection of the starch type is crucial to optimize starch-based materials’ properties and processing parameters for specific application requirements. 

## Figures and Tables

**Table 1:** Chemical composition of the starches and gelatin powders.

Material	Protein (wt.%)	Fat (wt.%)	Carbohydrates			Water (wt.%)	Ash (wt.%)
			Amylose (%)	Fiber (wt.%)	Total (wt.%)		
Gelatin	86.1	0.17	-	-	1.08	12.1	0.55
Native tapioca	0.21	0.18	26.4 ± 0.9	0.1	86.73	12.7	0.18
Pregelatinized tapioca	0.33	0.15	24.6 ± 0.4	2.6	87.56	11.8	0.16
Native corn	0.39	0.19	31 ± 1.2	1.3	86.35	12.9	0.17
Pregelatinized corn	0.69	0.74	35.7 ± 1.1	1.6	89.26	9.15	0.16

**Table 2:** Composition of the formulated liquids.

Sample	Water (wt.%)	Gelatin–Starch Solid Content (wt.%)	Gelatin (wt.%)	Starch (wt.%)	Starch Type
20 wt.% gelatin	75	20	20	-	-
25 wt.% gelatin	75	25	25	-	-
Native tapioca	75	25	20	5	native tapioca
Pregelatinized tapioca	75	25	20	5	pregelatinized tapioca
Native corn	75	25	20	5	native corn
Pregelatinized corn	75	25	20	5	pregelatinized corn

**Table 3:** Water content, average starch granule size, crystallinity degree and pattern of the four starch powders investigated.

Starch Type	Water Content (wt.%)	Average Granule Size (μm)	Crystal Pattern	Crystallinity Degree (%)
Native tapioca	12.7	17	C	36 ± 2
Pregelatinized tapioca	11.8	46	C	26 ± 1
Native corn	12.9	16	A	34 ± 1
Pregelatinized corn	9.15	71	- *	9 ± 2

* Predominantly amorphous.

**Table 4:** Gelatin and gelatin–starch suspensions density, surface tension, apparent viscosity and expansion ratio at 45 °C and gel strength.

Sample	Density of the Liquid (kg/m^3^)	Surface Tension at 45 °C (N/m)	Apparent Viscosity at 45 °C (mPa·s)	Expansion Ratio (mm/mm)	Gel Strength (N)
20 wt.% gelatin	1020 ± 20	31.24 ± 1.86	720 ± 42	13.66 ± 0.04	15.75 ± 0.27
25 wt.% gelatin	1060 ± 20	29.84 ± 1.79	1464 ± 141	10.93 ± 0.36	22.09 ± 0.74
Native Tapioca	1050 ± 10	39.28 ± 0.74	1768 ± 158	10.31 ± 0.41	14.51 ± 0.32
Pregelatinized Tapioca	1040 ± 20	36.80 ± 1.00	2066 ± 43	9.60 ± 0.55	17.87 ± 0.84
Native Corn	1070 ± 50	37.36 ± 0.58	2210 ± 67	9.64 ± 0.31	16.38 ± 0.42
Pregelatinized Corn	1120 ± 100	35.65 ± 1.97	2411 ± 98	8.88 ± 0.64	13.06 ± 0.32

**Table 5:** Comparison of the expansion ratio, bulk density, relative density and porosity of the foams.

Sample	Expansion Ratio (ER.)	Bulk Density (kg/m^3^)	Relative Density	Porosity (%)
20 wt.% gelatin	13.66 ± 0.04	28.58 ± 2.30	0.029	97.1
25 wt.% gelatin	10.93 ± 0.36	42.08 ± 0.63	0.042	95.8
Native Tapioca	10.31 ± 0.41	44.86 ± 2.64	0.046	95.4
Pregelatinized tapioca	9.60 ± 0.55	45.61 ± 1.87	0.045	95.5
Native Corn	9.42 ± 0.35	48.20 ± 1.23	0.042	95.8
Pregelatinized Corn	9.64 ± 0.31	49.45 ± 0.48	0.044	95.6

**Table 6:** Mean pore and cell areas of the foams with different formulations.

Sample	Mean Pore Area (mm^2^)	Mean Cell Area (mm^2^)
20 wt.% gelatin	0.098 ± 0.080	1.490 ± 1.228
25 wt.% gelatin	0.058 ± 0.040	0.775 ± 0.984
Native tapioca	0.058 ± 0.047	1.522 ± 2.413
Pregelatinized tapioca	0.039 ± 0.032	0.882 ± 0.875
Native corn	0.059 ± 0.067	1.351 ± 1.153
Pregelatinized corn	0.038 ± 0.033	0.839 ± 0.927

**Table 7:** Compression properties of gelatin, gelatin starch and EPS foams.

Sample	Bulk Density (kg/m^3^)	Compression Modulus (E*) (kPa)	Compression Strength (σ*_el_) (kPa)	Compression Strain (kPa)	Elastic Deformation Energy (kJ/m^3^)	Deformation Energy at 50% Strain (kJ/m^3^)
20 wt.% gelatin	28.58 ± 2.30	1.687 ± 100	62 ± 3	6.28 ± 0.61	2.60 ± 0.52	32.56 ± 2.03
25 wt.% gelatin	42.08 ± 0.63	4.062 ± 265	147 ± 11	6.45 ± 0.45	6.46 ± 1.41	80.60 ± 6.84
Native tapioca	44.86 ± 2.64	2.803 ± 453	117 ± 12	6.68 ± 1.43	4.54 ± 1.53	64.24 ± 3.83
Pregelatinized tapioca	45.61 ± 1.87	4.579 ± 688	160 ± 3	5.73 ± 0.35	6.34 ± 0.24	92.58 ± 8.19
Native corn	48.20 ± 1.23	4.929 ± 351	180 ± 8	5.03 ± 0.19	6.47 ± 0.29	98.60 ± 1.70
Pregelatinized corn	49.45 ± 0.48	4.181 ± 149	166 ± 3	6.16 ± 0.13	5.81 ± 0.35	86.63 ± 3.52
EPS 20 kg/m^3^	20 ± 1.03	1.932 ± 162	56 ± 2	- *	4.11 ± 0.12	38.96 ± 0.50
EPS 30 kg/m^3^	30 ± 1.26	4.102 ± 127	106 ± 2	- *	8.03 ± 0.17	67.30 ± 1.48

* Calculated from 10% strain.

## Data Availability

Data available on request.

## Supplementary Materials

The following supporting information can be downloaded at: https://www.mdpi.com/article/10.3390/polym15071775/s1. Figure S1. Optical microscopic pictures of (a) 20 wt.% gelatin, (b) 25 wt.% gelatin, (c) pregelatinized tapioca, (d) pregelatinized corn samples, (e) native corn samples, (f) native tapioca samples.
