# Supplementary material for: Effect of Starch Type and Pre-Treatment on the Properties of Gelatin–Starch Foams Produced by Mechanical Foaming
Source: Polymers (Basel). 2023 Apr 2;15(7):1775. doi: 10.3390/polym15071775 (PMC10096918; doi:10.3390/polym15071775)
Supplement: Supplementary file 1 [file polymers-15-01775-s001.zip › polymers-2184317-supplementary.pdf]

Supplementary

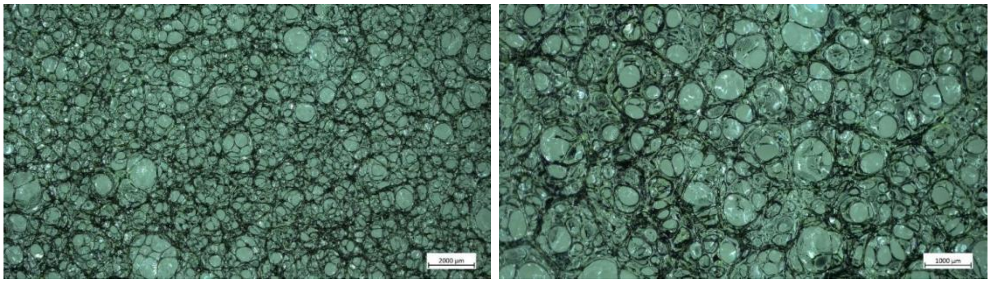

(a)

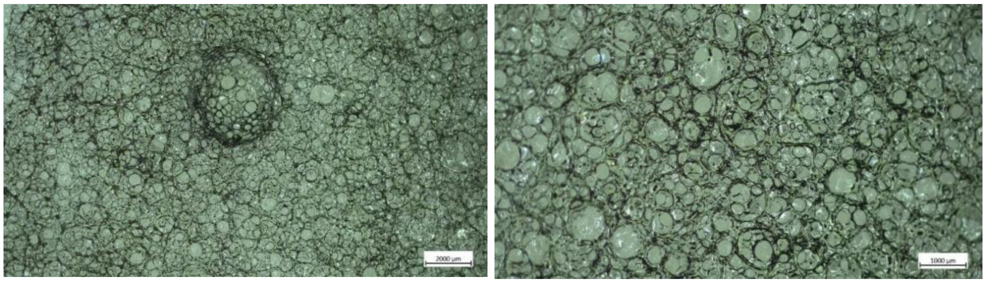

(b)

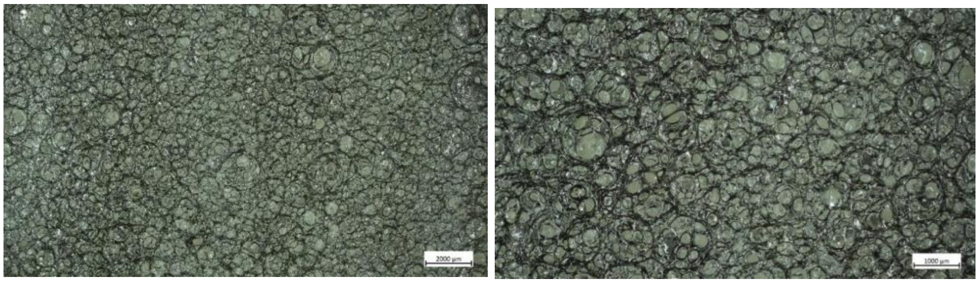

(c)

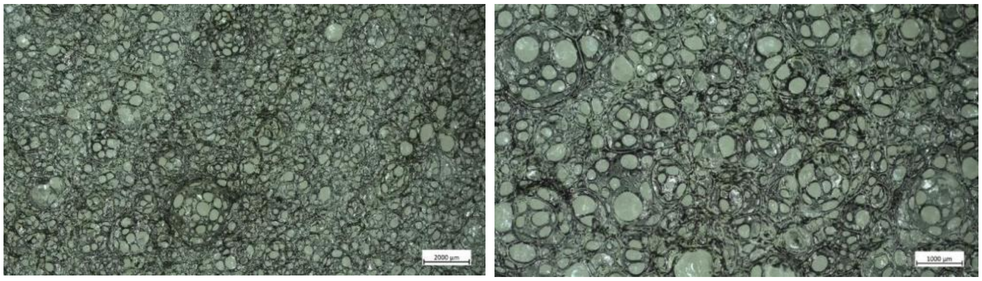

(d)

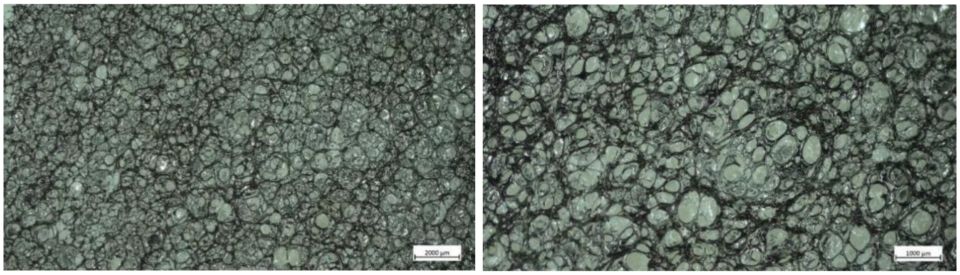

(e)

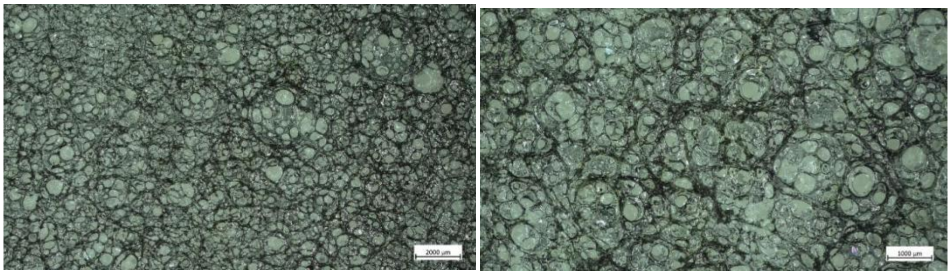

(f)

Figure S1. Optical microscopic pictures of (a) 20 wt.% gelatin, (b) 25 wt.% gelatin, (c) pregelatinized tapioca, (d) pregelatinized corn samples, (e) native corn samples, (f) native tapioca samples.
